# Position‐dependent offset corrections for ring applicator reconstruction in cervical cancer brachytherapy

**DOI:** 10.1002/acm2.70079

**Published:** 2025-03-19

**Authors:** Leon G. Aldrovandi, Matthias E. T. Dessein, Shelley M. Pearson, Shelley M. Bulling

**Affiliations:** ^1^ Radiation Treatment Wellington Blood and Cancer Centre Newtown Wellington New Zealand; ^2^ Varian Medical Systems Oncology House Crawley UK

**Keywords:** applicator commissioning, brachytherapy, quality assurance

## Abstract

**Purpose:**

Due to the tight curvature in their design, ring applicators are usually associated with large positioning errors. The standard practice to correct for these deviations based on global offsets may not be sufficient to comply with the recommended tolerance. In this work, we investigate two methods for applicator reconstruction that implement position‐dependent source offset corrections.

**Methods:**

Measurements were performed using the Varian Interstitial PEEK Ring 60° and a Varian BRAVOS afterloader. Source positioning was characterized by means of autoradiographs acquired for three different loading patterns and three ^192^Ir sources over a period of 5 months. Additionally, the actual source path was determined by means of a series of planar kV images for different dummy cable positions. The first position‐dependent correction method consists of locally modifying the radius of the reconstructed source path according to the measured offsets. The second method, recommended by Varian, simulates a bidirectional movement of the source during applicator reconstruction to compensate for positioning errors.

**Results:**

Autoradiographs showed a quasi‐linear increase of the dwell position offsets, with a negligible error at the tip and a value close to 3 mm at the end of the ring. This result, consistent with a circular wire movement with an effective radius 0.5 mm larger than the nominal value, was in agreement with the observations from the kV images. After implementation of the position‐dependent correction methods, residual positioning errors for the two methods resulted in a mean value (±1 SD) of 0.0 (±0.3) mm, and a range of [−0.7 mm, 0.7 mm].

**Conclusion:**

The two tested methods for applicator reconstruction with position‐dependent source offset corrections were able to successfully correct the positioning errors. The method recommended by the manufacturer had the additional advantages of a more straightforward implementation and the potential for use in other applicator types.

## 1 INTRODUCTION

Brachytherapy (BT) plays an integral role in the curative treatment of inoperable cervix cancer, enabling tumoricidal doses of radiation to be delivered directly to the primary site of disease.^1^ Several applicator designs are available for cervix cancer BT, with tandem/ovoid (T&O) reported as the most frequently used applicator (75% of cases) followed by the tandem/ring (T&R) (24% of cases).^2^ Applicator choice depends on several patient‐ and department‐related factors such as fit to patient anatomy, institutional historical practice, availability, and physician preference. From a dosimetric point of view, several studies have reported that for a given CTV_HR_ coverage the use of T&R applicators is associated with shorter treatment times, smaller treated volumes, lower rectum and bladder doses, but higher vagina 5 mm lateral‐point doses in comparison to the T&O applicator.^3^, ^4^, ^5^ Superior target dose and conformity with T&R applicators could be explained by the flexibility to activate source positions over a 360° angular space and the smaller build‐up caps (laterally and cranially) of the T&R design.^5^

The greater flexibility for planning and dosimetric benefits of the T&R comes at a cost, as the design of the ring applicators decreases the source positioning accuracy. Due to the tight ring curvature, the lumen diameter is usually required to be considerably larger than the source wire. The inherent tension, twisting, and flexing of the wire during its movement through the ring lumen causes the source to follow a complex path that deviates from the central lumen line.^6^ This “curving effect” may lead to source positioning errors of up to 6 mm.^7^, ^8^, ^9^, ^10^

In order to compensate for these errors and ensure that the dwell position accuracy meets the ±2 mm criterion stated by international recommendations,^6^, ^11^, ^12^ a thorough characterization and commissioning of every individual ring applicator must be performed prior to clinical use. The standard method to check source dwell positions is by means of the radiation of the active source, that is, autoradiographs. This can be carried out using film^9^, ^10^, ^13^, ^14^ or other high‐resolution detectors.^15^, ^16^, ^17^, ^18^ Acquisition of CT or MRI images of the applicator with marker wires in situ is an alternate method that is discouraged for curved applicators,^19^ as differences between the marker wire and source positioning of up to ±2.5 mm have been observed.^20^

Once the characterization of the ring applicator has been completed, the systematic misplacement of the source at every dwell position in the ring is known. The standard practice for correcting source positioning errors in rings is to derive a global offset for the applicator from the autoradiograph results, which is usually defined as the mean error across all dwell positions in the ring. Compensation for this global offset can be performed, for example, during applicator reconstruction in the treatment planning system (TPS).^8^ According to this correction method, the tip of the source channel is extrapolated distally beyond the actual ring inner tip by a distance equal to the calculated global offset. This results in a distal shift of the same magnitude to all dwells in the reconstructed applicator so that the dwell positions are correctly displayed in the planning CT or MR images. An alternative methodology for global offset compensation consists of modifying the delivery plan after import of the plan in the afterloader control software.^21^ For instance, in a Varian BRAVOS afterloader system, the Distal Position Correction tool can be used during treatment preparation to shift the dwell positions in a given channel by the same distance.^22^, ^23^

**FIGURE 1:**
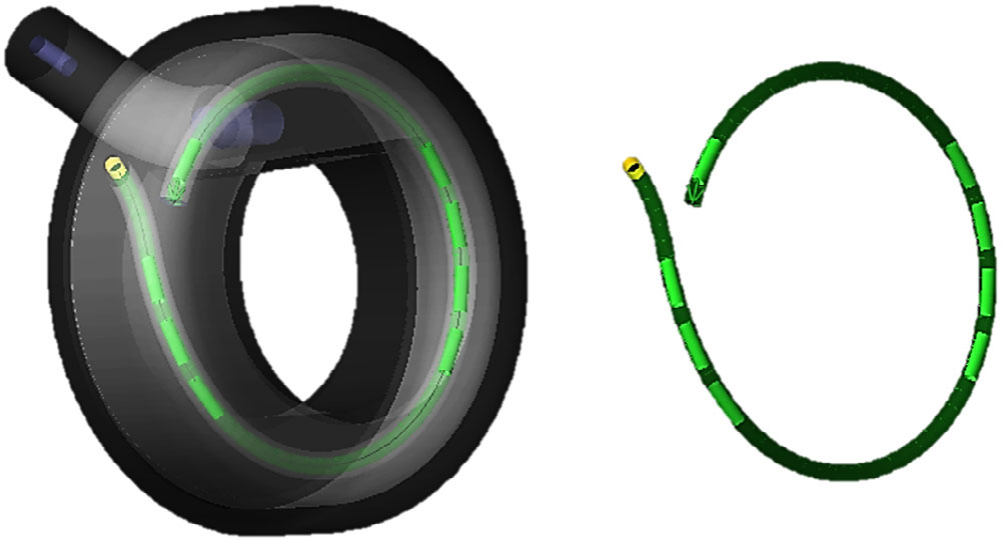
Design of the Varian PEEK ring used in this study. For an “ovoid‐like” loading pattern, the most distal and most proximal dwell positions are adjacent to each other.

However, the methods described above, based on applying the same global correction to all positions, present several limitations. A global offset may not be sufficient to comply with the ±2 mm criterion in cases of high variability in the magnitude of source misplacement at different dwell positions. Moreover, even if the commissioning results are within tolerance after the implementation of a global correction, positioning errors larger than 2 mm may appear during treatment delivery or routine applicator QA due to intrinsic afterloader repeatability, inter‐wire variability, and offset dependency with the treatment plan. Furthermore, the global approach may not be the most appropriate solution for certain Varian ring designs, like the PEEK ring model used in this study, in which the most distal and most proximal dwell positions are adjacent to each other for typical “ovoid‐like” loading patterns, as shown in Figure 1. Here positioning errors may lead to a change in the inter‐dwell distance between first and last dwell positions well outside tolerance that cannot be reduced by a global offset. With these limitations in mind, it is the purpose of the present work to investigate the benefits and clinical feasibility of two methods for ring applicator reconstruction that implement a local (i.e., dwell‐position dependent) source positioning correction.

## 2 METHODS

The applicator used in this study was the ring probe 60° GM11010500 (lot #U23) from the 3D Interstitial Ring Applicator Set 60° GM11010190 (Varian Medical Systems Inc., Palo Alto, California, USA). This ring is made of polyether ether ketone (PEEK) and has a nominal (source path) diameter of 30 mm and a nominal lumen width of 2 mm. All the measurements were performed using a BRAVOS v1.2 afterloader system (Varian Medical Systems Inc., Palo Alto, California, USA) equipped with a GammaMed Plus HDR ^192^Ir source (3.5 mm long active core, 0.9 mm cable diameter).^24^ A Ring applicator component type was used in BRAVOS in all cases. In addition, the same 1000 mm transfer guide tube GM11010870 (Varian Medical Systems Inc., Palo Alto, California, USA) was used, resulting in a nominal channel length of 132 cm. Afterloader quality assurance checks performed before every measurement session included source and dummy cable positional accuracy with the BRAVOS CamScale device and BRAVOS channel length measurement accuracy using a decommissioned tandem applicator AL07522000.

### 2.1 kV imaging analysis

In order to determine the actual path followed by the source, an analysis of the positioning of the cable inside the lumen was performed by acquiring a series of kV images with a dummy cable placed at different positions. We chose to use the same model of dummy cable in the afterloader so that stiffness and twisting of the cable was similar to the active wire. To simulate the way the active cable is positioned by the afterloader, the dummy cable was first inserted up to the lumen tip and then manually retracted to the remaining positions. Cable retraction between consecutive images was approximately 5 mm, and a total of 25 images were acquired to cover the whole length of the ring. Images were acquired on a TrueBeam linear accelerator (Varian Medical Systems Inc., Palo Alto, California, USA) with 100 cm source‐ring distance, 150 cm source‐imager distance, 50 kV, 40 mA, 40 ms exposure time, and small focal spot.

In‐house software was developed using MATLAB R2022a (The MathWorks, Inc., Natick, Massachusetts, USA) to process the digital images in order to filter the position of the cable in each image, and identify the inner and outer surface of the internal lumen. Based on this information, quantitative data regarding the position of the wire tip and the center of the radiative source was extracted for every position of the cable. Additionally, the images allowed assessment of wire repositioning within the ring lumen during cable retraction.

### 2.2 Solid model validation

The solid model for the GM11010500 applicator was installed in the Solid Applicator library of Eclipse Brachytherapy Planning v16.1 TPS (Varian Medical Systems Inc., Palo Alto, California, USA) and validated against the physical applicator. The solid model is required in this work for autoradiograph analysis.

For validation, a high‐resolution CT image of the applicator was acquired using a Siemens Somatom Confidence RT Pro scanner (Siemens Healthineers, Erlangen, Germany), with a slice thickness of 0.50 mm, an axial resolution of 0.14 mm, and the applicator stem aligned longitudinally on the scanner couch. The solid model was registered to the CT images in Eclipse using the ring surface as reference. In particular, rotational alignment in the ring plane (i.e., around the ring axis), critical for source position identification, was determined using the straight section present in the inner side of GM11010500 rings. To quantify the discrepancy between the solid model and the real applicator, the applicator was manually reconstructed by defining the source path centered on the applicator lumen visible in the CT imaging, while the applicator tip was identified using the Line Profile tool provided by the TPS. An in‐house software was developed in MATLAB to calculate the distance between dwell positions when the same loading pattern (equidistant with 5 mm inter‐dwell distance) was used in both manually reconstructed and solid model applicators. The difference in lumen tip position between solid model and manually reconstructed applicator, of relevance for autoradiograph analysis, was also measured during this comparison.

### 2.3 Autoradiographs

In our institution, autoradiograph tests for GM11010500 rings are performed following a three‐step process. First, a piece of RTQA2 Gafchromic film (Ashland, Wilmington, Delaware, USA) is taped to the ring, and the film is exposed using a HDR plan with dwell times of 2 s for a nominal 10 Ci ^192^Ir source. During the second step, the film remains taped to the applicator and is exposed with an electron beam (6 MeV, 100 MU, 100 cm SSD) from a TrueBeam linear accelerator (for further film‐solid model registration). In the last step, the film is digitized at 150 dpi using an Epson Expression 10000XL flatbed scanner (Seiko Epson, Suwa, Nagano, Japan), imported into Eclipse TPS and registered to the solid model. The registration, film‐solid model is performed based on the ring geometry visible in the film image (from the electron beam) and is required to determine the position of the lumen tip. Consistent with the CT‐solid model registration done during solid model validation, rotational alignment around the ring axis is determined by using the straight section of the ring as reference. Any discrepancy in tip position between solid model and the real applicator identified during solid model validation must be considered and corrected at this point. Once the position of the lumen tip is known, the angle from tip to each dwell position is measured using the TPS Measure Angle tool. The corresponding offset ε_i_ at the i‐th dwell position p_i_ can then be calculated from the equation:

(1) $$\varepsilon_{i}=R_{i}\left(\theta_{\mathrm{plan},i}-\theta_{\mathrm{meas},i}\right)$$

where:

θ_plan,i_: angle (in radians) from ring lumen tip to source center at i‐th dwell position p_i_ measured in TPS reconstructed applicator

θ_meas,i_: angle (in radians) from ring lumen tip to source center at i‐th dwell position p_i_ measured in autoradiograph

R_i_: ring radius at i‐th dwell position p_i_ (to a good approximation the nominal radius can be used here)

It can be seen from Equation 1 that a positive offset means a delivered source position located more distally (i.e., closer to the tip) than planned. This will be the convention used throughout this work.

Since the offset for each dwell position may depend on the HDR plan, films were exposed with three different loading patterns. Two plans had an equidistant loading (1 cm inter‐dwell distance) with first dwell position at 1 and 6 mm from the tip, respectively. The third plan corresponded to a “clinical” loading that followed the standard institutional practice with dwell positions replicating a typical ovoid geometry. Moreover, to take into account the possible dependence of source positioning with the active wire, measurements were performed for three different GammaMed Plus HDR source wires. In total, eight autoradiographs were acquired over a period of 5 months.

It is worth mentioning that during the Treatment Preparation step, completed before the exposure of each autoradiograph, BRAVOS performed a channel length measurement with the dummy cable. In the same way as is done in clinical plans, in case of deviations between the planned channel length and the BRAVOS measured channel length, the afterloader updated the channel length and adjusted the dwell positions to maintain the planned distances to the tip. The planned channel length is defined in the TPS during plan generation and was determined in our case at the time of applicator commissioning using the Measurement Ruler AL13169000 (Varian Medical Systems Inc., Palo Alto, California, USA). In addition, all plans were delivered using a Ring component type in BRAVOS v1.2 control software. In contrast to just flexible and rigid types available in previous versions, BRAVOS version 1.2 implements four applicator component types: flexible, needle, rigid, and ring. For each component type, the control software takes the geometry and other characteristics of the component into account when measuring the channel length and positioning the source.^25^

### 2.4 Position‐dependent correction methods

#### 2.4.1 Method 1 – Variable source‐path radius

The first method to correct a position‐dependent offset involves modifying the radius of the source path at a given point of the ring according to the measured offset in that sector. Let us suppose the planned (i.e., TPS‐reconstructed) and actual source trajectories are determined by the radii *R*_pl_ (θ) and *R*_act_ (θ), respectively. In each case, the source path length at a given angle θ can be determined by:

(2) $$\begin{array}{ccc} \mathrm{path}\;{\mathrm{length}}_{a}\left(\theta \right) & =\int_{\theta_{0,a}}^{\theta }\sqrt{R_{a}^{2}+{\left(\frac{{\mathrm{dR}}_{a}}{d\theta }\right)}^{2}d\theta^{\prime }} & \\ & \approx \int_{\theta_{0,a}}^{\theta }\left(R_{a}+\frac{1}{2R_{a}}{\left(\frac{{\mathrm{dR}}_{a}}{d\theta }\right)}^{2}\right)d\theta^{\prime }\; & a=\mathrm{pl},\;\mathrm{act} \end{array}$$

where the assumption of a slowly varying radius dR_a_/dθ < < *R*_a_ (expected to be satisfied by a realistic ring reconstruction) was made to obtain the last expression.

If we define the offset ε at a given angle θ as

(3) $$\varepsilon \;\left(\theta \right)={\mathrm{path}\;\mathrm{length}}_{\mathrm{act}}\;\left(\theta \right)-{\mathrm{path}\;\mathrm{length}}_{\mathrm{pl}}\left(\theta \right)$$

then at the i‐th dwell position (with planned and actual angles θ_plan,i_ and θ_meas,i_, respectively), the offset can be calculated from:

(4) $$\begin{array}{ccc} \varepsilon \left(\theta_{\mathrm{meas},i}\right) & \approx & \int_{\theta_{\mathrm{meas},i}}^{\theta_{\mathrm{plan},i}}\left(R_{\mathrm{pl}}+\frac{1}{2R_{\mathrm{pl}}}{\left(\frac{{\mathrm{dR}}_{\mathrm{pl}}}{d\theta }\right)}^{2}\right)d\theta^{\prime }\\ & & \approx R_{\mathrm{pl},i}\left(\theta_{\mathrm{plan},i}-\theta_{\mathrm{meas},i}\right) \end{array}$$

where the assumptions of θ_plan,i_ ≈ θ_meas,i_ and slowly varying radius were used. Note that in this way we recover the offset expression given in Equation 1. Once the offset for each one of the N dwell positions is determined from Equation 4, the offset ε at any angle θ can be estimated, for instance, by means of spline interpolation or curve fitting.

In order to correct the offsets ε, the radius of the TPS‐reconstructed ring needs to be locally modified by a quantity Δ*R* = *R*_act_—*R*_pl_. By using Equations 2 and 3, it can be shown that ΔR must satisfy the following non‐linear first‐order differential equation:

(5) $$\Delta R+\frac{1}{R_{\mathrm{pl}}}\frac{d\Delta R}{d\theta }\;\left(\frac{{\mathrm{dR}}_{\mathrm{pl}}}{d\theta }+\frac{1}{2}\frac{d\Delta R}{d\theta }\right)=\frac{d\varepsilon }{d\theta }\;$$

where a small change in radius (i.e., Δ*R*<<*R*_a_) and a slowly varying radius (dR_a_/dθ<<*R*_a_) were assumed.

Note that error compensation for the first position can always be performed by offsetting the definition of the reconstructed channel tip from the real lumen end by an amount equal to the offset ε(θ_1_). After this correction, the initial value for Equation 5 will be Δ*R*(θ_1_) = 0.

It is also worth noticing that if the offset ε can be considered as linearly increasing between two given dwell positions, then Δ*R* can be approximated in that sector by:

(6) $$\Delta R\approx \frac{R_{\mathrm{pl}}.\Delta \varepsilon }{d}$$

where *R*_pl_ is the mean planned radius in that sector, Δε is the change in offset between dwell positions, and *d* is the inter‐dwell distance.

Although Equation 5 can, in principle, be solved numerically to obtain Δ*R* for all angles, a semi‐graphical approach can be used in the Eclipse TPS which may result in a more straightforward implementation of the methodology. An initial correction of the ring radius can be estimated from Equation 5 just by assuming Δ*R* = dε/dθ. After applying this correction to the reconstructed ring, any significant residual offset can be graphically corrected in the TPS by dragging the contour points (i.e., the points defining the source path) in the radial direction until the source angular positions in the TPS agree with those derived from the autoradiograph.

#### 2.4.2 Method 2 – Bidirectional source movement

An alternative method for position‐dependent correction of the source positioning errors in ring applicators is presented by Varian in two videos available in VarianThink under the title Ring Commissioning—Bravos Afterloader. This method consists of simulating a bidirectional movement of the source in the inter‐dwell intervals to compensate for the positioning errors. In Eclipse, a bidirectional source movement can be generated at the time of applicator reconstruction by dragging a contour point towards the applicator tip until it overpasses the preceding contour point. For instance, if the contour point CP_i_ is dragged over the previous point CP_i‐1_ so that it is now in a more distal position, the length of the source path will be calculated in Eclipse as if the source would move proximally (retract) up to CP_i‐1_, then change the direction of movement to move distally (extend) up to CP_i_, and after that move proximally again towards CP_i+1_. Given that this bidirectional movement is an artifice generated to correct positioning errors, it is important not to define any dwell position in that sector (that is, between contour points CP_i‐1_ and CP_i+1_ in the example given before). It is also worth mentioning that no information regarding contour points is transferred to the afterloader, then the treatment plan is not affected by these changes in the TPS, and the source will maintain the standard unidirectional movement and dwell positions during treatment delivery.

A fundamental limitation of this method is that it only allows to reduce the distance between planned dwell positions reconstructed in the TPS, but not to increase it. If the difference between offsets at two consecutive positions p_i‐1_ and p_i_ is Δε_i_ = ε_i_—ε_i‐1_, only cases with Δε_i+1_ ≥ Δε_i_ ≥ 0 (i.e., with measured inter‐dwell distances smaller than planned and monotonically decreasing with the distance to tip) can be completely corrected through this method. A wider range of cases can be addressed by combining this local method with a tip redefinition global correction method. Finding the optimal solution in this case requires solving an optimization problem subject to inequality constraint functions. A description of the optimization problem for the general case and the corresponding solution that minimize the distance between planned and delivered dwell positions is presented in the Appendix. We have developed an in‐house MATLAB script to solve the optimization problem in the general case, which is provided as Supplementary Material.

## 3 RESULTS

### 3.1 kV imaging analysis

**FIGURE 2:**
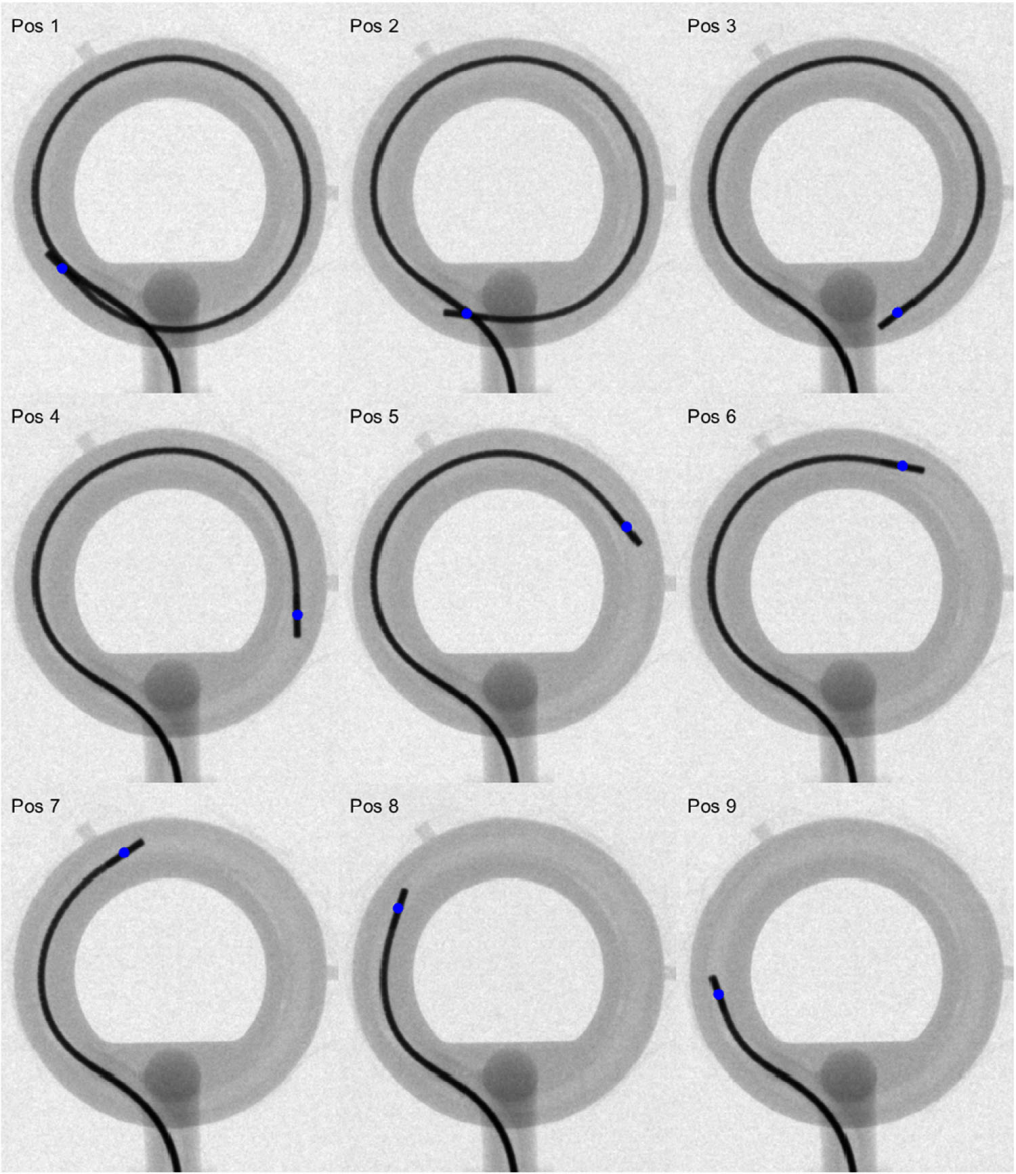
Subset of the 25 kV images of the ring, acquired on a TrueBeam linear accelerator with an inactive wire at different positions. The blue circles represent the center of the source, which is located at 2.5 mm from the wire tip for BRAVOS afterloaders.

**FIGURE 3:**
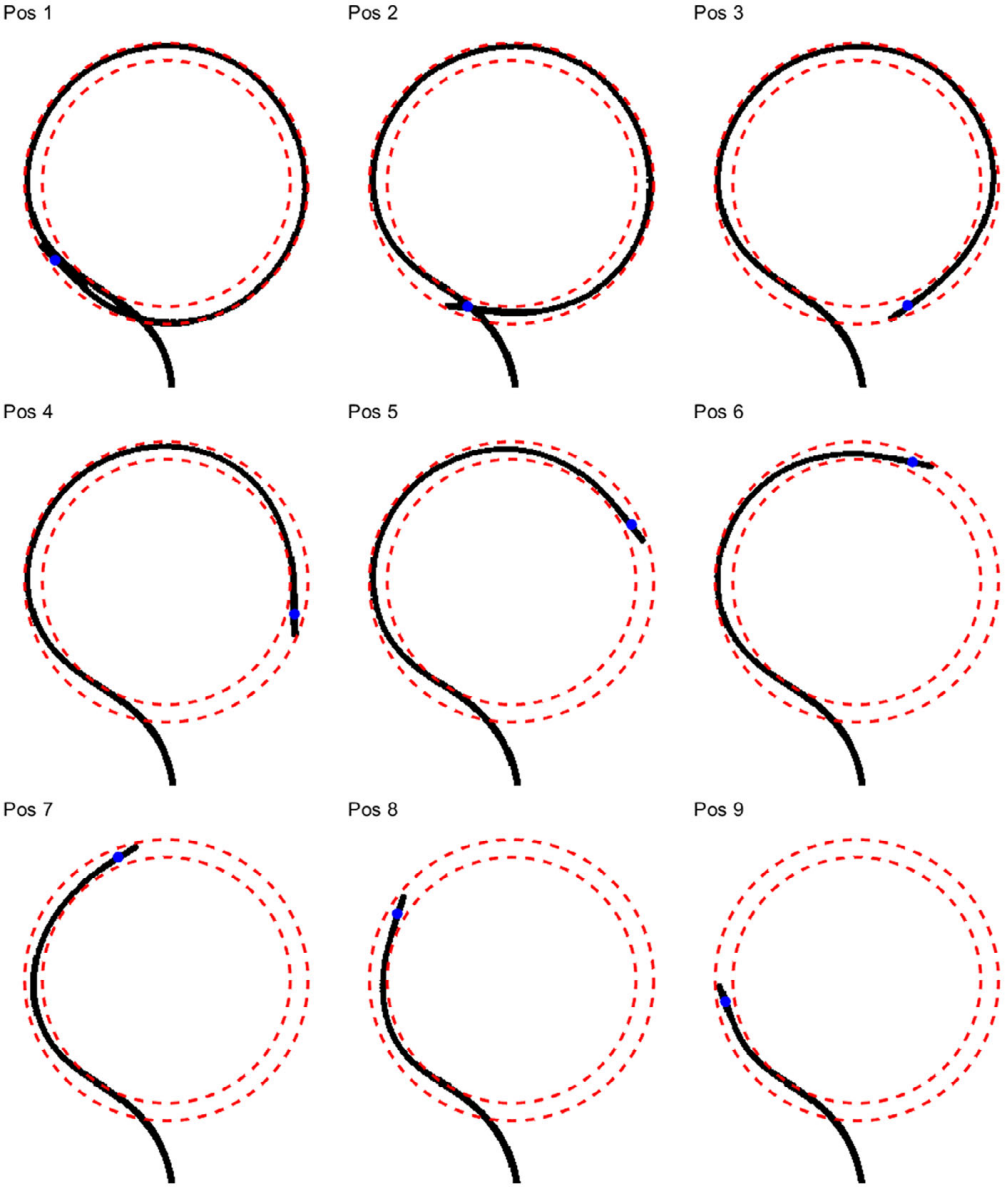
Wire position filtered from kV images shown in Figure 2. Inner and outer surfaces of the lumen for a nominal ring radius are displayed as red dashed lines. It can be seen that the wire tip is always in contact with the outer lumen surface. Moreover, large portions of the wire are also in close contact with the outer surface except for the most proximal dwell positions. Based on these images, an effective radius of the source path larger than the nominal value would be expected.

A subset of the 25 kV images of the ring applicator with an inactive wire at different positions is shown in Figure 2. The position of the center of the source, located at 2.5 mm from the wire tip for BRAVOS afterloaders, was determined through a MATLAB in‐house software and is represented in Figure 2 by blue circles. To obtain a better understanding of the position of the wire inside the ring lumen, the information of the wire was extracted from the images by using the aforementioned software. Figure 3 displays the wire for every image in Figure 2 along with the inner and outer lumen surfaces for a nominal ring radius.

**FIGURE 4:**
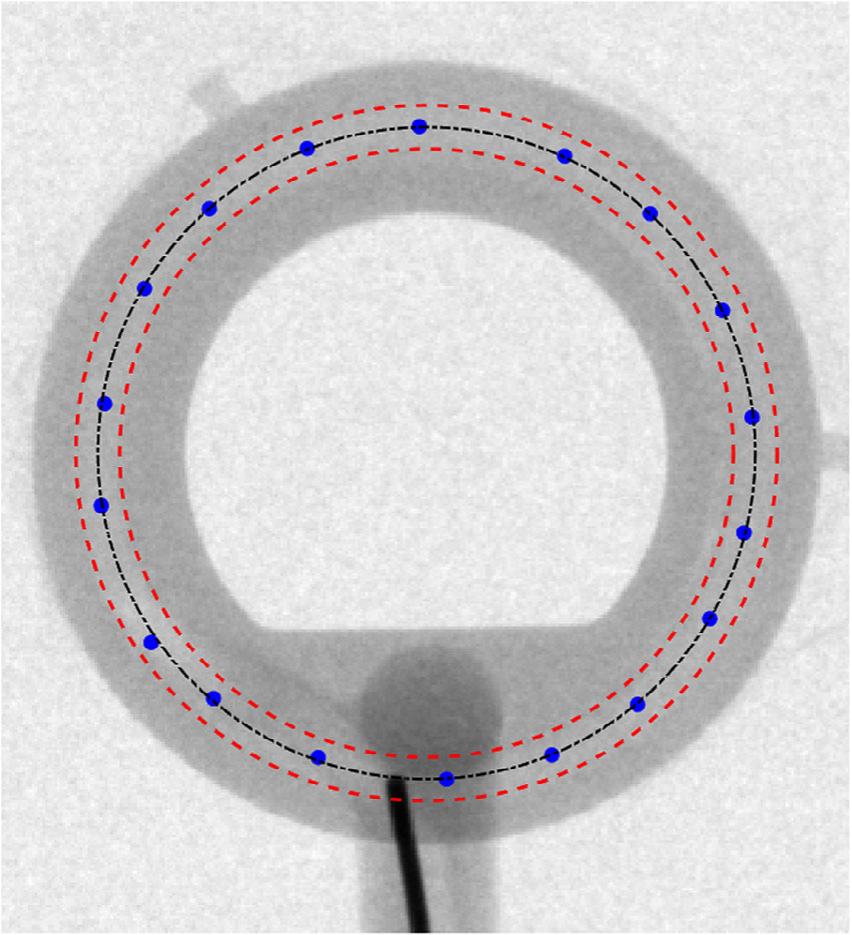
Position of the source center derived from the kV ring images are shown as blue circles. Red dashed lines represent the inner and outer surfaces of the lumen, while the black dash‐dotted line is the central axis of the lumen for a nominal ring radius. It can be verified that the average positions of the source center agree with the lumen axis.

Several conclusions can be derived from Figures 2 and 3 with respect to the cable positioning inside the ring lumen:

• The wire tip is in contact with the outer surface of the lumen throughout the entire movement in the ring.
• Intrinsic stiffness of this source wire type causes the most distal section (approximately 10 mm length) of the cable to remain straight and does not follow the curved geometry of the applicator.
• The first consequence of the previous point is that, even though the wire tip is in contact with the outer surface, the actual position of the source is closer to the lumen center. In fact, a more quantitative analysis of the source positions derived from the kV ring images reveals that on average the source center is at exactly 15 mm from the ring center, a distance that matches the nominal ring radius as can be seen in Figure 4.
• Another effect resulting from the initial straight section of the cable is that the source axis is not tangent to the source path as standardly assumed during applicator reconstruction in the TPS. However, an analysis of the dosimetric consequences of this effect is beyond the scope of this work.
• Large portions of the wire are in close contact with the outer surface of the lumen for most of the kV images. This means that the effective radius of the actual source path is expected to be larger than the nominal radius, in agreement with previous literature.^10^

### 3.2 Solid model validation

**FIGURE 5:**
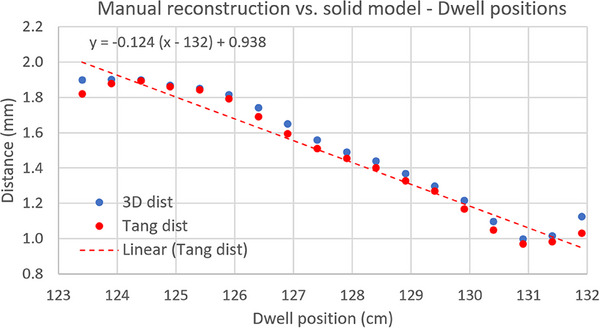
Distance between dwell positions from CT‐based manual direct reconstruction and Varian solid model. Blue dots represent the Euclidean distance between 3D coordinates of each pair of dwell positions, while red dots correspond to the tangential component of the distance (i.e., distance along an axis tangent to the source path). The quasi‐linear increase in the distance can be considered as an indication that the physical ring has a larger diameter than the nominal value from the solid model.

**FIGURE 6:**
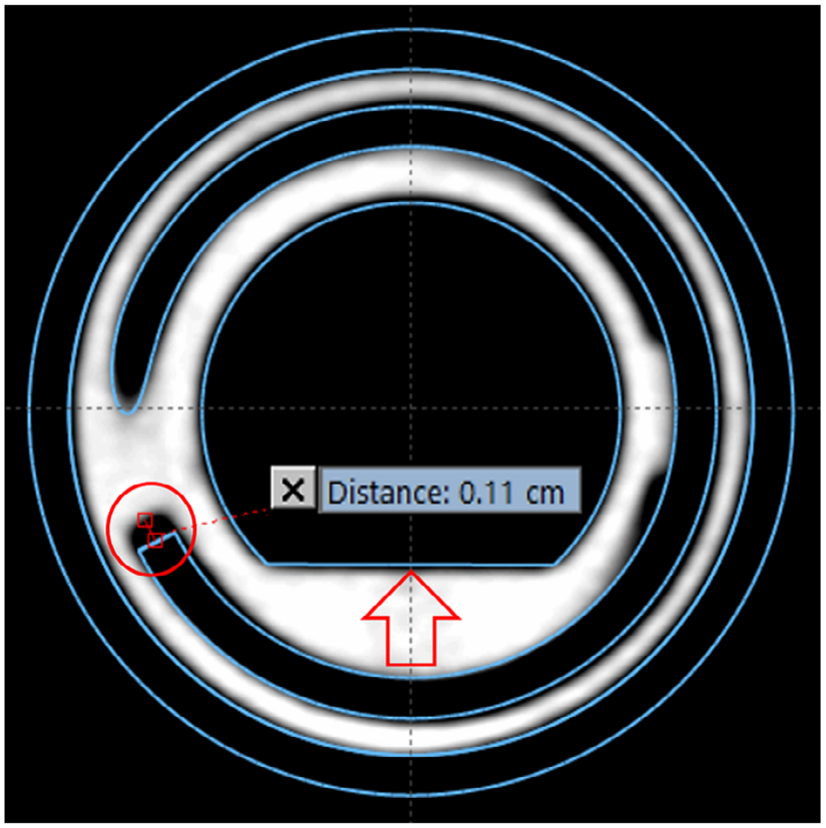
Registration between CT images and solid model was done based on the outer surface of the ring. In particular, angular orientation was determined by matching the straight part of the applicator (marked with a red arrow). A discrepancy of about 1 mm in the position of the lumen tip (marked with a red circle) is clearly visible in the image.

**FIGURE 7:**
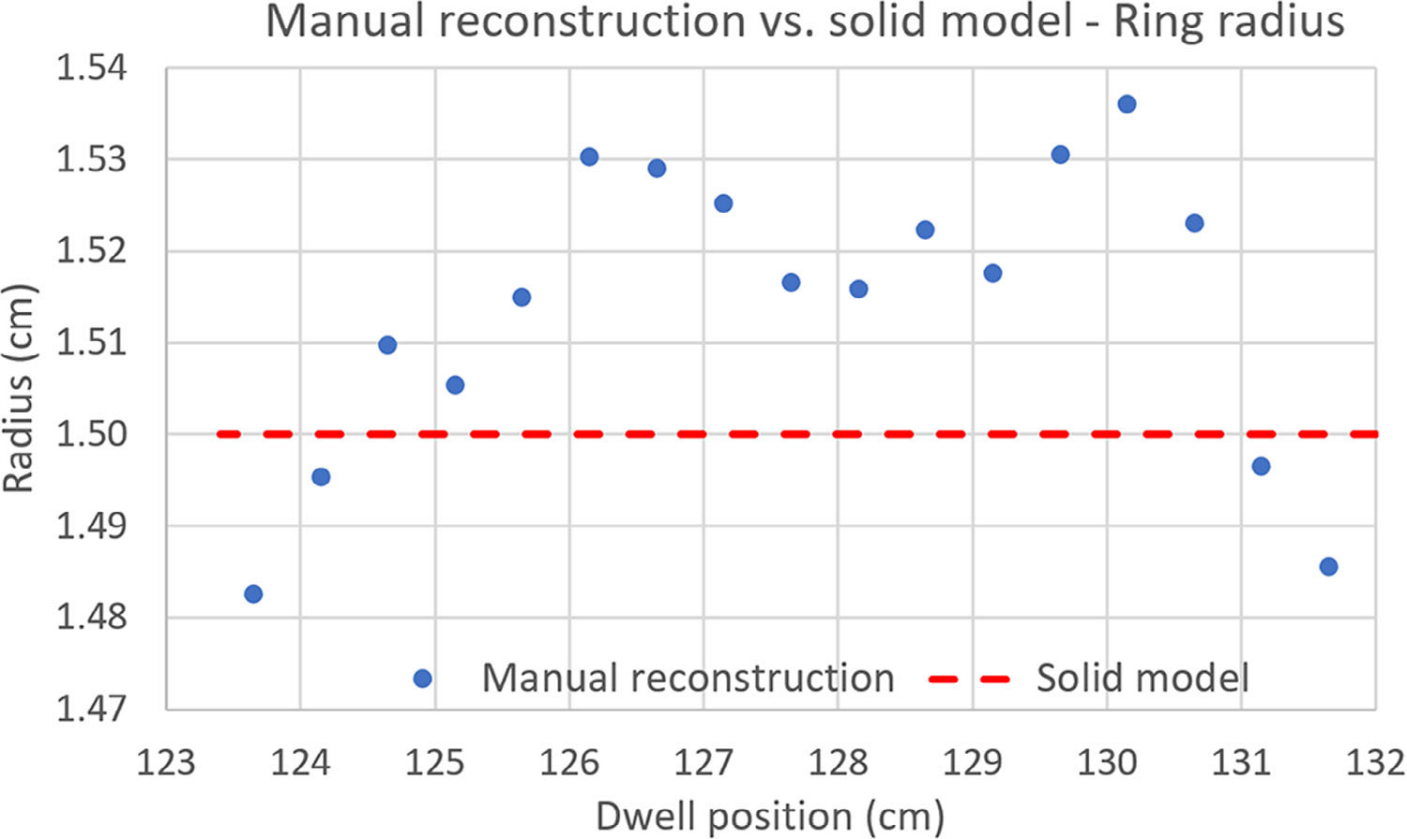
Radius of the physical ring for each inter‐dwell sector (blue dots), measured on CT images from ring center to lumen axis, and nominal ring radius from the solid model (red dashed line). It can be seen that the actual radius of the ring used in this study is on average 0.15 mm larger than the nominal value. This is consistent with the linear increase in the distance between dwell positions observed in Figure 5.

The 3D coordinates of the dwell position from a plan based on the Varian solid model were compared with those from a plan based on manual direct reconstruction. For a clearer interpretation of the results, two distances between each pair of dwell positions were calculated: the total 3D distance and the tangential distance (i.e., distance along an axis tangent to the source path). Results are shown in Figure 5, and some conclusions can be drawn from this graph:

- There is a discrepancy of about 1 mm in the first dwell position. This is due to a difference of the same magnitude in the position of the lumen tip (red circle in Figure 6) when solid model and CT image are registered based on the straight part of the applicator (marked with a red arrow in Figure 6)
- As expected, non‐tangential contribution to the 3D distance is negligible.
- A quasi‐linear increase in the distance between dwell positions is evident from the linear fit of the data in Figure 5. This linear growth can be considered as an indication that the physical ring has a larger diameter than the nominal value from the solid model. The effective radius for each inter‐dwell sector was estimated using Equation 6 and plotted in Figure 7. These values were then verified to coincide (within ± 0.1 mm) with the radius (distance from ring center to lumen center) measured in the CT image set. It is interesting to note that an average difference in ring radius as small as 0.15 mm produces an error in source positioning of 1 mm at the most proximal dwell positions.

### 3.3 Autoradiographs

**FIGURE 8:**
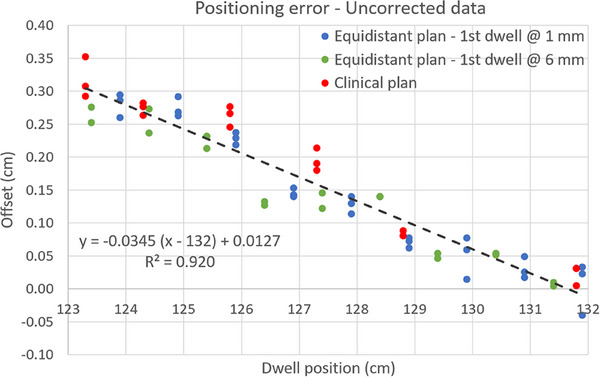
Dwell position offsets for the eight autoradiographs acquired with three different loading plans and three different sources over a period of 5 months. From the linear fitting of all data points, it can be concluded that for the ring used in this work the offset at the tip is negligible, while for the subsequent positions the offset is consistent with an effective circular source path with 15.5 mm radius.

Offsets calculated from Equation 1 for each dwell position in the eight autoradiographs acquired with the GM11010500 ring are shown in Figure 8. The combined data from all plans can be fitted with reasonably low residual values (± 0.7 mm) by a linear model. As it will be discussed in the next section, the linear increment in the offsets can be attributed to a source following an effective circular path with a radius larger than the 15.0 mm nominal value for this ring model.

Despite the noticeable inter‐plan variation in the offsets, no clear correlation could be found between the measured offsets and the corresponding loading pattern. Regarding the dependency with the active wire, the variation in source positioning for a given plan was similar (about 0.2 mm on average) when autoradiographs acquired with the same or different active wires were compared. Then, the inter‐wire variation in the offsets can be deemed negligible for the three wires included in this study.

Interestingly, the channel length measurement performed by BRAVOS during the Treatment Preparation stage resulted in a length value of 131.8 cm for all autoradiographs. On the other hand, the nominal channel length of 132.0 cm was confirmed during ring commissioning using the Measurement Ruler AL13169000, and this value was used in the TPS during plan generation. Due to this difference between the planned and BRAVOS measured channel length, BRAVOS control software adjusted the dwell positions immediately before treatment delivery to be 2 mm more proximal than initially planned. Note that, although three different active wires were used to acquire the autoradiographs, the same dummy wire was used during BRAVOS channel length measurements as the dummy wire is replaced annually in our institution.

### 3.4 Position‐dependent correction methods

For the implementation of the two position‐dependent correction methods, we began with a reconstruction of the applicator centered on the internal lumen visible in the high‐resolution CT image previously acquired for the GM11010500 ring.

**FIGURE 9:**
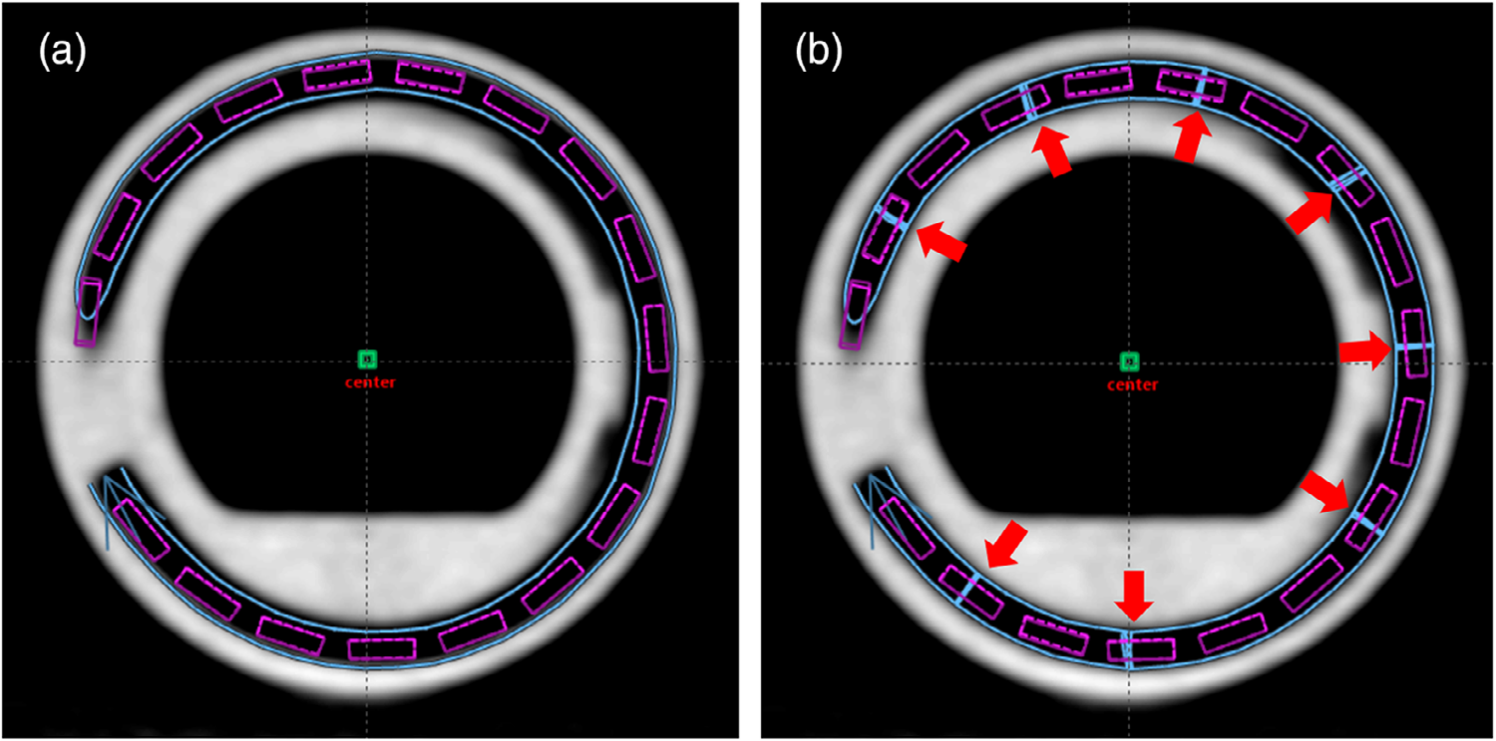
(a) Source path corresponding to the “Variable source‐path radius” method. The radius of the trajectory at each point was calculated using Equation 5 and the offsets from linear fitting in Figure 8. (b) Source path from the “Bidirectional source movement” method. Sectors of bidirectional movement are marked with red arrows.

As described earlier, for the “Variable source‐path radius” method, the radius of the trajectory at each point was calculated using Equation 5 and the offsets from data fitting in Figure 8 (see Figure 9a). In this case, a linear fit of the offsets implies that ΔR is constant for all angles and then Equation 5 can be exactly solved resulting in an effective ring radius of 15.5 mm. Interestingly, the difference of approximately 0.5 mm between nominal and effective radius is consistent with the 0.9 mm diameter wire mostly moving along the outer surface of the 2 mm diameter lumen, in agreement with the information derived from Figures 2 and 3.

On the other hand, for the “Bidirectional source movement” method, sectors of bidirectional movement were defined at convenient inter‐dwell positions (red arrows in Figure 9b) to have all planned source positions agreeing with the positions derived from the linear fitting.

**FIGURE 10:**
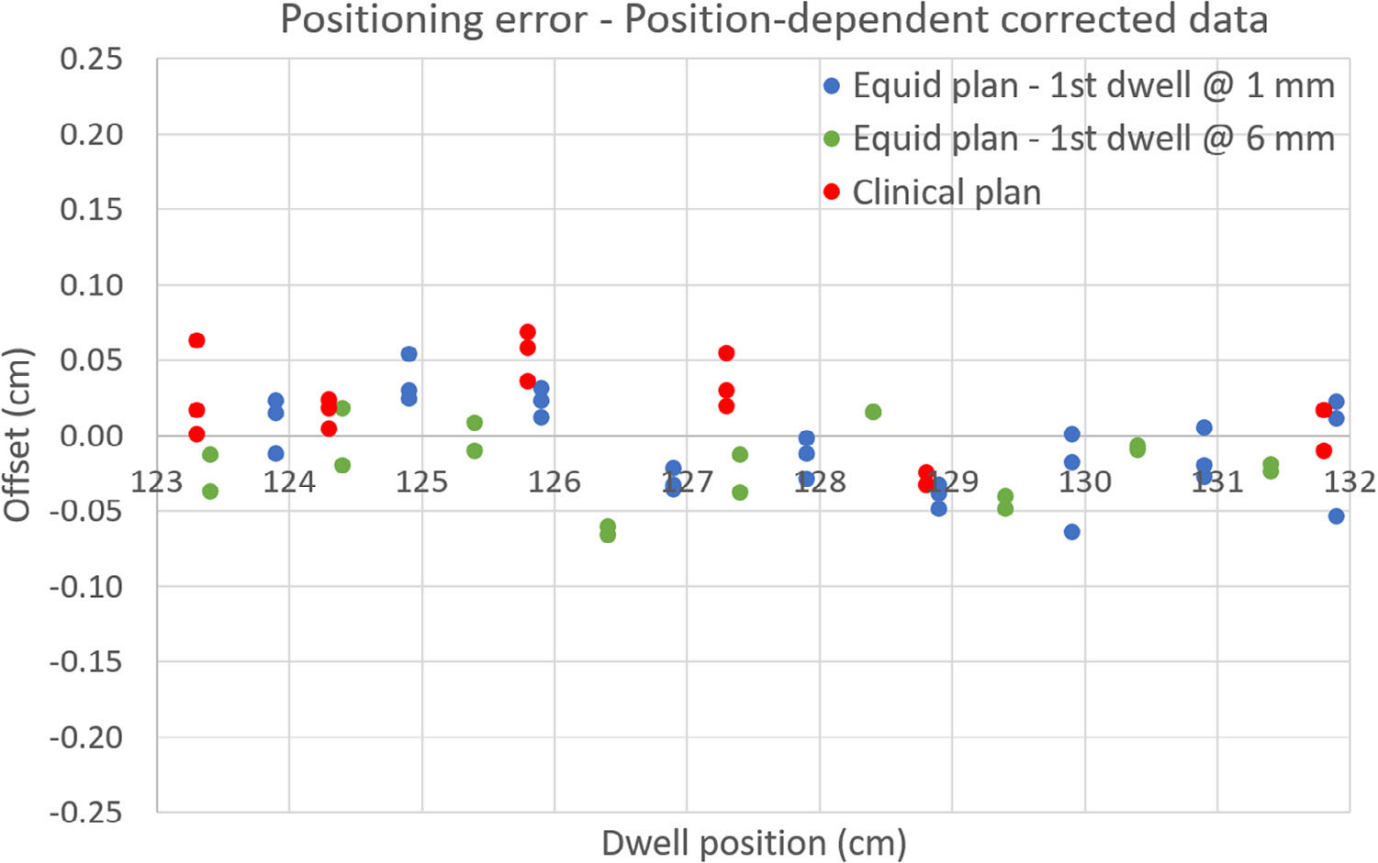
Residual offsets for all data points from eight autoradiographs after the implementation in the TPS of the position‐dependent offset correction. Both correction methods analyzed in this study lead to the same planned angular position for the dwell positions, and therefore, to identical residual errors. Mean error (± 1 SD) and range is 0.0 (± 0.3) mm and [‐0.7 mm, 0.7 mm], respectively.

In both cases, corrections were done following a distal‐to‐proximal order. As expected, the final angular position of each planned dwell coincides for both methods. The residual offsets for the eight autoradiographs resulting from the corrected source paths are shown in Figure 10. Mean error (± 1 standard deviation) and error range for the full set of data points are: 0.0 mm (± 0.3 mm) and [‐0.7 mm, 0.7 mm].

## 4 DISCUSSION

The measurement of the offset for each dwell position is a crucial step during the commissioning of a ring applicator. Although the standard practice is to acquire the autoradiograph with an equidistant loading pattern (usually, with a 1 cm inter‐dwell distance),^21^ evaluation of source position for more “clinical” plans may contribute to a better characterization of wire movement and source positioning in the ring. Moreover, measurements with a given plan should be repeated several times for the same applicator to account for source position repeatability and to detect intermittent problems in source positioning such as those reported in the literature.^16^, ^26^

A thorough validation of the corresponding solid models must also be included as part of the applicator commissioning if they are intended to be used clinically for applicator reconstruction or during the autoradiograph process as in the present work. In our case, when the solid model was registered to the applicator CT images based on the outer ring surface, we found a discrepancy in lumen tip position of 1 mm (Figure 6). Furthermore, a mean increase as small as 0.15 mm in the radius of the reconstructed source trajectory (manually reconstructed centered in the ring lumen) with respect to the nominal 15 mm value led to an additional error of 1 mm. Both effects yield the differences in dwell positions between the solid model and the manually reconstructed ring shown in Figure 5, with a maximum value close to 2 mm. Given the magnitude of these errors, they should be accounted for at the time of using the solid model and, preferably, the source path linked to the solid model should be redefined to match the actual applicator geometry whenever the tools of the TPS allow to do that.

The set of kV images acquired to estimate the actual source path suggests that the wire tip is mostly in contact with the outer surface of the ring lumen but that, due to the most distal section of the cable remaining straight, the actual source center (located at 2.5 mm from the wire tip) follows a trajectory closer to the lumen center (Figures 2 and 3). In addition, it was observed in the images that large portions of the wire are in close contact with the outer surface of the lumen, indicating that the effective radius of the actual source path may be larger than the nominal radius. Although these conclusions are consistent with the results from the autoradiographs (see below) and with previous literature,^10^, ^27^ it is worth mentioning that the kV analysis performed in this work is affected by some limitations. First, the dummy wire was manually retracted between dwell positions, which may result in a wire tension and source trajectory different to when the wire is driven by the afterloader. In addition, since the cable retraction and kV image acquisition was performed only once, this analysis does not consider variations due to intrinsic lack of afterloader positioning repeatability. Due to the same reason, inter‐wire and inter‐plan variations in source positioning were not analyzed. Last but not least, differences between active and dummy wires may also affect the results.

The analysis of the set of eight autoradiographs acquired for the ring showed a quasi‐linear increase in the value of the dwell position offsets, starting from a negligible error at the tip and reaching a value close to 3 mm at the end of the ring (Figure 8). This behavior can be interpreted as the wire following a circular path with an effective radius of 15.5 mm, that is, moving along the outer surface of the lumen. This is in close agreement with the conclusions drawn from the kV images acquired of the ring with an inactive wire at different source positions (Figures 2 and 3). Note that the mean offset value across all dwell positions (Figure 8) for the ring studied in this work is approximately 1.5 mm. Fagerstrom presented the results from the characterization of seven Varian PEEK rings, including two rings of the same model used in the present work.^23^ The author found mean offset values varying between 4 and 5 mm for all seven rings. The reason for the discrepancy between our results and those from Fagerstrom is not clear to us, but it might be due to differences in the version of the afterloader control software. As mentioned before, BRAVOS version 1.2 implements new applicator component types that take into account the applicator geometry and affect both channel length measurement and source positioning during treatment delivery.^25^

It is also worth mentioning that in this work we used Equation 1 for offset calculation during the autoradiograph analysis, which differs from the manufacturer's recommendation given in the Instruction for Use documentation.^21^ The methodology recommended by Varian requires calculating the path length at a given dwell position from:

(7) $$\mathrm{Path}\;\mathrm{length}\;=\left(\pi \;r\;\frac{\alpha }{180^{\circ }}\right)-\mathrm{SCDT}$$

where *r* is the nominal ring radius, α is the angle between the tip of the lumen and the center of the radiation source footprint, and SCDT is the source center distance to the tip of the source cable (2.5 mm for a BRAVOS afterloader). However, Equation 7 may introduce an error of several millimeters in the calculated path length of the most proximal dwell positions if the effective radius of source trajectory differs from the nominal radius.

Once the offset at each dwell position is known, a common practice is to apply a systematic shift to all dwell positions by a distance equal to the mean offset value (close to 1.5 mm in our case). This can be done, for instance, by redefining the tip of the reconstructed ring during applicator reconstruction in the TPS or by modifying the delivery plan after import of the plan in the afterloader control software. However, positioning errors after implementation of this global correction may still exceed the ±2 mm tolerance during treatment delivery or routine applicator QA due to factors such as intrinsic afterloader repeatability and offset variability for different treatment plans, different active wires or wire usage history; all factors that cannot be totally accounted for during ring commissioning. Moreover, the offsets displayed in Figure 8 are particularly problematic for ring designs similar to that from the model used in this work (GM11010500). With this ring design, the most distal and most proximal dwell positions are adjacent and heavily loaded in plans replicating an “ovoid‐like” pattern (Figure 1), typically used to protect organs at risk (OARs) such as rectum and bladder. As a result, the 3 mm error in inter‐dwell distance between these two positions cannot be corrected by a global correction method.

Complementary to the global offset correction, another approach recommended by Varian in the Instruction for Use documentation to reduce source positioning errors consists of having a dwell at the most distal position (1 mm from ring lumen tip in the BRAVOS afterloader) with the minimum dwell time allowed by the afterloader software.^21^, ^23^ Thus, the first position is only used to move the source cable to the most distal position but does not deliver a clinically relevant dose. After retraction of the cable to the second dwell position the cable is tightened which improves equidistant movement of the cable for the following positions. However, this pull‐back methodology was not considered in this work as it significantly reduces the dwell positions available when an “ovoid‐like” pattern (see Figure 1) is required to protect OARs. Moreover, the changes implemented in the last version of the BRAVOS control software appear to have reduced considerably the bunching between first and second dwell positions observed in previous versions of the afterloader.^22^

The two methods evaluated in this work for local correction of source positioning errors, named here as “Variable source‐path radius” and “Bidirectional source movement” methods, proved to be capable of compensating for the offsets shown in Figure 8. In both cases, the post‐correction residual positioning errors have a mean value of 0.0 mm, a range of [‐0.7 mm, 0.7 mm] and a standard deviation of 0.3 mm (see Figure 10). The magnitude of the residual errors can be considered as low if we take into account that these values correspond to data acquired from eight autoradiographs with three different plans and three different sources, and compares favorably against previously published measurement uncertainties.^26^, ^28^

On the other hand, both methods present some limitations worth mentioning. In the case of the “Bidirectional source movement” method, its main limitation lies in the fact that it only allows to correct the reconstructed ring in the TPS by displacing the dwell positions more distally (closer to the lumen tip), but not more proximally. In other words, it is not possible to correct local offsets when the measured inter‐dwell distance is larger than planned. To compensate for this limitation, the “Bidirectional source movement” method can be combined with a tip redefinition global correction method. The optimal set of local corrections in this combined approach can be found by solving an optimization problem subject to inequality constraints as described in detail in the Appendix.

Regarding the “Variable source‐path radius” method, although this method corrects offsets of any sign, the magnitude of the correction that can be reached may be insufficient if a realistic source path is desired. Since the radial position of the actual source trajectory cannot be determined from autoradiographs with sufficient accuracy, the radial error (i.e., the distance in the radial direction between reconstructed and actual source trajectories) is usually unknown. In order to have a radial error within acceptable values, it is convenient to keep the reconstructed source trajectory inside the true lumen section of the ring. Then, for a lumen diameter of 2 mm, the change in radius from the central lumen axis is restricted to be smaller than 1 mm. By using the simplified expression given by Equation 6, we can see that the maximum offset than can be locally corrected every 10 mm of source path will be smaller than 0.7 mm (assuming a ring of nominal radius of 15 mm, like the one used in this study).

Based on the limitations described above and the experience gathered during this work, it was decided at this institution to opt for the “Bidirectional source movement” method (combined with the tip redefinition global method) as the preferred reconstruction methodology for ring applicators. Some of the advantages of the “Bidirectional source movement” method over the “Variable source‐path radius” method are:

• The implementation is easier. The optimization problem providing the optimal correction at each dwell position needs to be solved just once as the respective equations depend only on the measured offsets, and not on applicator characteristics.
• Use in other ring designs. Due to the limitations in the magnitude of local corrections that can be implemented with the “Variable source‐path radius” method, the “Bidirectional source movement” method may result in lower residual errors in other ring designs. Preliminary testing at our institution has confirmed that this is the case for open‐ended Varian Titanium rings.^8^, ^26^
• The reconstructed path can be defined centered in the ring lumen, most likely resulting in lower radial positioning errors.
• The methodology can be easily extended to other applicator types. Although offset corrections in applicators other than rings are not usually needed,^22^, ^23^ the “Bidirectional source movement” method can be used with any type of applicator geometry.

In order to facilitate the clinical implementation of local correction methods in Eclipse TPS, a suggested step‐by‐step approach to be followed as part of ring commissioning is described below:

1. Applicator imaging: Acquire CT images of the applicator with narrowest possible reconstructed slice width (preferably about 0.5 mm or smaller), small reconstructed Field of View (e.g. close to 10 cm) and a sharp kernel to increase the image resolution.
2. Initial applicator reconstruction: In the TPS, perform a manual direct reconstruction of the applicator by defining the source path centered on the applicator lumen visible in the CT imaging. The Line Profile tool available in Eclipse can be used to identify the lumen tip.
3. Solid model validation: In Eclipse, register the solid model with the actual ring as displayed in the high‐resolution CT imaging. It is important at this point to perform the registration based on the same ring references to be used in clinical cases during the registration between solid model and patient CT or MR images (typically, external ring surfaces, applicator stem, and/or needle holes in the case of interstitial rings). After solid model registration, verify agreement between solid model and actual ring. Note that comparison between source paths from manual reconstruction and from solid model is not needed for the next steps as only the manual reconstruction is used. However, discrepancies between the tips of both source paths must be identified at this stage if the solid model is used during autoradiograph analysis to locate the tip of the ring lumen.
4. Source position characterization: Determine the actual source positioning in the ring by means of autoradiograph verification. To have a more reliable characterization, acquire multiple autoradiographs, preferably with both equidistant and “clinical” loading patterns as well as different source wires (e.g., by acquiring autoradiographs immediately before and after a source exchange^23^). Autoradiograph analysis requires the measurement of the angle subtended from the tip of the applicator lumen to the center of each radiation source's footprint. The methodology used to locate the tip of the ring lumen in the film may vary depending on the ring geometry (e.g., whether ring is open‐ended or closed‐ended). But if solid models are used in this process, any discrepancy between solid model and actual ring tip identified in the previous step must be included in the film analysis.
5. Offset calculation: Use Equation 1 to calculate the offset at each dwell position from the angles measured in Eclipse TPS and autoradiographs between ring lumen tip and source center. For TPS angle measurements, use the initial manual reconstruction of the applicator, while for autoradiographs use the average angle across all films.
6. Final applicator reconstruction: The initial source path reconstruction can be corrected in the TPS by using either of two local correction methods. As discussed above, the “Bidirectional source movement” method is probably the most convenient approach. In this case, the correction required at each dwell position can be obtained from the calculated offsets by using the in‐house MATLAB script provided as Supplementary Material.
7. Plan Template generation: The final step consists in the creation of a Plan Template. In the plan with the solid model registered to the CT ring images created during solid model validation, delete the applicator but keep the solid model. Copy in this plan the applicator with the final reconstruction from the previous step and group both applicator and solid model. Finally, save the plan as a Plan Template.

For plan generation of patient treatments, the Plan Template created during ring commissioning is inserted in the patient CT or MR study, and the solid model is then registered to the applicator in the patient images based on the same ring references used at the time of solid model validation. Note that the use of a Plan Template avoids the need to implement offset compensation methods for each patient (either at planning or at treatment preparation stages), as the Plan Template's source path already incorporates the required position corrections.

A word of caution is warranted here as the standard methodology for applicator reconstruction review (usually consisting of verifying the tip definition and the alignment of the source path with the lumen axis) may not be applicable and/or sufficient when the position‐dependent correction methods have been used. In our institution, an Eclipse ESAPI script has been developed to automatically verify that the distance between every pair of subsequent contour points coincides with that of the template, guaranteeing in this way that no contour point has been inadvertently displaced. This has been done by using the Shape property of the Catheter class, which provides accessibility from ESAPI to the contour point coordinates.

## 5 CONCLUSION

Discrepancies in the TPS solid model and differences between the nominal and effective source path radius were found to be contributors to the disagreement between planned and delivered source positions. Using the nominal radius in the TPS leads to quasi‐linear dwell position errors that increase with distance from the lumen tip. The average effective radius was 0.5 mm larger than the nominal radius, resulting in a 3 mm error at the most proximal dwell.

Both the “Variable source‐path radius” and “Bidirectional source movement” methods for position‐dependent offset correction were able to successfully correct the measured source positioning errors, with mean (± 1σ) and maximum residual errors of 0.0 (± 0.3) and 0.7 mm, respectively. Moreover, the latter method, recommended by the manufacturer, was found to offer additional advantages such as a more straightforward implementation and the potential use in other applicator types.

## AUTHOR CONTRIBUTIONS

Leon Aldrovandi: Study conception and design, data collection, analysis and interpretation of results, and writing of the manuscript. Matthias Dessein: Study design, interpretation of results, review and edition of the manuscript. Shelley Pearson: Clinical input to the project, interpretation of results, review and edition of the manuscript. Shelley Bulling: Study design, supervision and coordination, review and edition of the manuscript. All the authors reviewed the final manuscript and approved the submission.

## CONFLICT OF INTEREST STATEMENT

No conflicts of interest.

## Supporting information



Supporting Information

## Optimum correction for “Bidirectional source movement” method

Let us suppose the case of N dwell positions whose offsets (obtained from Equation 1) are given by {ε_1_, …, ε_N_}. Based on these offsets, the reconstructed dwell positions will be corrected in the TPS by a magnitude equal to {δ_1_, …, δ_N_}. Here a correction δ_i_ > 0 means that the i‐th dwell position is reconstructed δ_i_ units more distally (i.e., toward the lumen tip) than the initial planned position. Then, a set of corrections {δ_i_} will be optimal if it minimizes the objective function F:

(A1)
$$F\;\left({\;\delta \;\;\;}_{1},\;\;\;\;\;\text{…},\;\;\;\;\;{\;\;\;\delta \;\;\;}_{N}\right)=\frac{1}{2}\underset{i\;\;\;\;\;=\;\;\;\;\;1}{\sum^{N}}\;{\left({\;\;\;\delta \;\;\;}_{i}-{\;\;\;\varepsilon \;\;\;}_{i}\right)}^{2}$$

When a correction in a given dwell position is performed in the TPS, the corresponding change in the position of contour points will also generate a displacement of the same magnitude for all subsequent (more proximal) dwell positions. Then, assuming that the correction in dwell positions is implemented in the TPS in a distal‐to‐proximal order, it is more appropriate to express the corrections in terms of a new set of variables {x_1_, …, x_N_} that gives the correction needed at each dwell when the corrections at previous (more distal) dwells have already been implemented. This new set {x_1_, …, x_N_} is given by:

(A2)
$$\begin{array}{c} x_{1}=\delta_{1}\\ x_{i}=\delta_{i}-\delta_{i-1}\;i=2,\text{…},N \end{array}$$

Note that by redefining the tip of the reconstructed applicator, the value of x_1_ can take any sign. However, since the subsequent dwell positions are corrected by means of the “Bidirectional source movement” method, these positions can only be displaced towards the lumen tip. As a result, the variables {x_2_, …, x_N_} are subject to the N‐1 inequality constraints:

(A3)
$$x_{i}\geq 0\;i\;=2,\;\text{…},\;N$$

By expressing the variables {δ_i_} in terms of variables {x_i_} as:

(A4)
$$\delta_{i}=\sum_{j=1}^{i}x_{j}\;i\;=1,\text{…},N\;$$

the objective function F defined in Equation A1 now takes the form:

(A5)
$$F\;\;\;\;\;\left(x_{1},\;\;\;\;\;\text{…},\;\;\;\;\;x_{N}\right)=\frac{1}{2}\underset{i\;\;\;\;\;=\;\;\;\;\;1}{\sum^{N}}\;{\left(\underset{j\;\;\;\;\;=\;\;\;\;\;1}{\sum^{i}}\;x_{j}-{\;\;\;\varepsilon \;\;\;}_{i}\right)}^{2}$$

where the variables {x_i_} are subject to the inequality constraints given in Equation A3.

According to the Karush‐Kahn‐Tucker (KKT) conditions,^29^ if {x_1_, …, x_N_} is a local minimizer of F, then exists a set of N‐1 KKT multipliers {µ_2_, …, µ_N_} such that

(A6)
$$\begin{array}{cc} \frac{\partial }{\partial x_{i}}\left(F\left(x_{1},\text{…},x_{N}\right)-\sum_{j=2}^{N}\mu_{j}\cdot \;x_{j}\right) & \\ =\sum_{j=i}^{N}\left(\sum_{k=1}^{j}x_{k}\;-\varepsilon_{j}\right)-\mu_{i}\cdot \theta \left(i-2\right)=0 & \;i=1,\text{…},N\\ x_{i}\geq 0 & \;i=2,\text{…},N\\ \mu_{i}\geq 0 & \;i=2,\text{…},N\\ \sum_{j=2}^{N}\mu_{j}\cdot \;x_{j}=0 & \end{array}$$

where θ(n) is the discrete Heaviside step function:

(A7)
$$\theta \;\left(n\right)=\left\{\begin{array}{c} 0,\;n<0\\ 1,\;n\geq 0 \end{array}\right.\;$$

## Appendix

Using the relation satisfied by the objective function F

(A8)
$$\frac{\partial F}{\partial x_{i}}-\;\frac{\partial F}{\partial x_{i+1}}=\sum_{j=1}^{i}x_{j}\;-\varepsilon_{i}$$

it can be shown that the first equation in Equation A6 is equivalent to

(A9)
$$\sum_{j=1}^{i}x_{j}=\varepsilon_{i}+\mu_{i}\theta \left(i-2\right)-\mu_{i+}\theta \left(i-1\right)\;i=1,\text{…},N$$

Since

(A10)
$$\begin{array}{c} \sum_{j=1}^{i}x_{j}=x_{i}+\sum_{j\;=1}^{i-1}x_{j}=x_{i}+\varepsilon_{i-1}+\mu_{i-1}\theta \left(i-3\right)-\mu_{i}\theta \left(i-2\right) \end{array}$$

the first equation in Equation A6 can finally be expressed as

(A11)
$$\begin{array}{ccc} & & x_{i}-2\;\mu_{i}\;\theta \left(i-2\right)+\mu_{i+}\;\theta \left(i-1\right)+\mu_{i-}\;\theta \left(i-3\right)\\ & & \;=\varepsilon_{i}-\varepsilon_{\text{i-1}}\;i=1,\text{…},\;N \end{array}$$

On the other hand, the last three equations in Equation A6 imply that for each i ≥ 2 either the constraint is active (x_i_ = 0) or the corresponding KKT multiplier is null (µ_i_ = 0). This means that there are 2^N‐1^ possible local minimizers of F. Since each of these candidates has x_i_ = 0 or µ_i_ = 0 for every i ≥ 2, Equation A11 will in these cases reduce to a set of *N* equations with *N* unknown variables. After solving Equation A11 for each one of the 2^N‐1^ candidates, the optimal solution will be that satisfying the constraints {x_i_ ≥ 0, µ_i_ ≥ 0, i = 2, …, N} and resulting in a minimum value of the objective function F.

An in‐house MATLAB script that provides the optimal set of corrections {x_1_, …, x_N_} based on the methodology described above is given as Supplementary Material.
